# *Pleurotus eryngii* Mushrooms Fermented with Human Fecal Microbiota Protect Intestinal Barrier Integrity: Immune Modulation and Signalling Pathways Counter Deoxycholic Acid-Induced Disruption in Healthy Colonic Tissue

**DOI:** 10.3390/nu17040694

**Published:** 2025-02-14

**Authors:** Evangelia N. Kerezoudi, Georgios I. Zervakis, Vasiliki Pletsa, Adamantini Kyriacou, Robert J. Brummer, Ignacio Rangel

**Affiliations:** 1 Nutrition-Gut-Brain Interactions Research Centre, School of Medical Sciences, Örebro University, 70182 Örebro, Sweden; robert.brummer@oru.se (R.J.B.); ignacio.rangel@oru.se (I.R.); 2 Department of Nutrition and Dietetics, Harokopio University, 17676 Athens, Greece; kyriacou@hua.gr; 3 Laboratory of General and Agricultural Microbiology, Department of Crop Science, Agricultural University of Athens, 11855 Athens, Greece; zervakis@aua.gr; 4 Institute of Chemical Biology, National Hellenic Research Foundation, 11635 Athens, Greece; vpletsa@eie.gr

**Keywords:** *Pleurotus eryngii* mushrooms, deoxycholic bile acid, gut barrier, Ussing chamber, cytokines, signalling pathway

## Abstract

**Background:** This study explores the potential of the *Pleurotus eryngii* mushroom fermentation supernatant (FS-PEWS) as an intervention for mitigating sodium deoxycholate (SDC)-induced intestinal barrier dysfunction and inflammation. **Methods**: FS-PEWS was assessed for its protective effects against SDC-induced barrier dysfunction and inflammation using an *in vitro* Caco-2 cell model and *ex vivo* colonic biopsies from healthy adult donors, where barrier integrity, permeability, immunomodulation and receptor-mediated pathways were evaluated. **Results:** In Caco-2 cells, SDC exposure downregulated *ZO-1*, *occludin*, and *claudin-1* expression, with FS-PEWS restoring *ZO-1* and *claudin-1* levels while maintaining cell viability. In colonic biopsies from healthy adults, FS-PEWS maintained tissue integrity and selectively mitigated transcellular permeability without affecting paracellular permeability when combined with the stressor. Additionally, FS-PEWS exhibited potent anti-inflammatory effects, reducing pro-inflammatory cytokines, e.g., *TNF-α*, *IL-6*, and *IL-1β* and modulating receptor-mediated pathways, i.e., *TLR-4*, *dectin-1*. **Conclusions:** These results demonstrate the potential of FS-PEWS to sustain intestinal barrier function and modulate immune responses under stress, highlighting its therapeutic potential for managing gut barrier dysfunction and inflammation associated with microbial metabolite-induced disruptions.

## 1. Introduction

The gastrointestinal (GI) tract constitutes the most expansive and complex interface between the internal environment of the host and the external milieu, serving as both a site for nutrient absorption and a critical barrier against external insults. This interface operates as a highly regulated bidirectional permeability system, where intestinal homeostasis—defined by a balanced interaction between the intestinal epithelium, resident microbiota and host immune responses—plays a pivotal role in maintaining systemic health [1]. Under physiological equilibrium, the intestinal barrier must precisely manage the balance between selective permeability for the absorption of dietary nutrients from the intestinal lumen into the systemic circulation and internal milieu and the defensive mechanisms that inhibit the penetration of pathogens and harmful external agents [2]. In order to achieve this, the intestinal barrier is responsible for regulating substance transfer via both transcellular passage (through the cell), under the presence of selective transporters of molecules and paracellular passage (between the cells), which occurs between adjacent cells and is governed by the permeability of the intercellular junctions [3]. This functional unit strictly depends on the integrity of the gut epithelium, supported by junctional proteins, such as tight junctions (TJ), desmosomes, and adherent junctions, which form a physical barrier and connect adjacent epithelial cells, together with the lamina propria [4]. 

Barrier dysfunction often arises from a combination of inflammatory mediators, bacterial endotoxins such as lipopolysaccharides (LPS) and cytotoxic agents that destabilize junctional integrity [5]. A growing body of evidence highlights the detrimental role of secondary bile acids that are linked to westernized dietary patterns in this process. For example, following the intake of lipid-rich meals, the concentration of bile acids in the colon can rise to nearly 1 mM [6]. Among these, deoxycholic acid (DCA) is predominantly produced by the microbial transformation of primary bile acids, exacerbating intestinal barrier dysfunction by inducing oxidative stress, inflammation and apoptosis of epithelial cells [7]. This destabilization leads to increased intestinal permeability—often referred to as “leaky gut”—allowing the translocation of pathogens, antigens, and toxins into the systemic circulation. These translocated molecules can elicit local and systemic immune responses, involving pro-inflammatory cytokines such as tumour necrosis factor-alpha (TNF-α), interleukin-6 (IL-6), and interleukin-1β (IL-1β), which further contribute to the disintegration of the epithelial barrier [8,9,10,11]. The detrimental effects of DCA extend beyond inflammatory signalling, as it also compromises epithelial cell viability by inducing mitochondrial dysfunction and oxidative stress, leading to apoptotic cell death. Moreover, its amphiphilic nature disrupts cell membrane integrity, weakening epithelial cohesion and further exacerbating gut permeability defects. Mechanistically, DCA has been shown to activate the TLR-4/NF-kB signalling pathway, amplifying inflammation-driven barrier dysfunction by promoting the upregulation of cytokines such as TNF-α and IL-6 [12,13]. As a result, a wide range of intestinal and extra-intestinal pathological conditions, such as celiac disease, intestinal ischemia, Inflammatory Bowel Diseases (IBDs), Irritable Bowel Syndrome (IBS), food allergies, Type I Diabetes, obesity, Parkinson’s disease, depression, autism, and asthma have been linked to gut barrier’s disintegration [14,15,16,17,18,19,20,21].

The preservation of intestinal barrier integrity is intricately tied to the diversity and functionality of the gut microbiota. Dysbiosis, characterized by a disruption in the composition and metabolic activity of the microbial ecosystem, is considered a critical factor in the onset of barrier dysfunction and associated pathological conditions [22]. Rebalancing the microbiota has been shown to restore barrier integrity, largely through enhanced fermentation of dietary substrates, leading to increased production of short-chain fatty acids (SCFAs), particularly acetate, propionate and butyrate—the predominant metabolic end products [23]. Diet plays a pivotal role as a major environmental determinant of microbiota composition and function [24]. A diet rich in fibre and prebiotics—“substrates that are selectively utilized by host microorganisms conferring a health benefit”—can stimulate the proliferation of beneficial bacteria in situ [25]. Latest data support that among other prebiotic-rich functional foods, *Pleurotus eryngii* mushrooms possess properties that contribute to the functional enhancement of a disturbed intestinal barrier [26,27,28]. Notably, *P. eryngii* is a rich source of dietary fibre (27.5 g/100 g), polysaccharides (6.85 g/100 g), and protein (19.15 g/100 g), with *β*-glucans representing the predominant fibre fraction (38.7–49.7% *w*/*w*) [28,29]. These *β*-glucans remain largely intact after *in vitro* digestion, indicating their availability for microbial fermentation and supporting their prebiotic potential [28]. Beyond its prebiotic properties, *P. eryngii* has been shown to exert direct effects on mucosal immunity and inflammatory signalling pathways [30,31]. The bioactive polysaccharides and secondary metabolites of *P. eryngii* play a role in maintaining epithelial integrity by modulating tight junction dynamics [15,16,19]. Additionally, *P. eryngii* has been demonstrated to attenuate inflammation by inhibiting NF-kB activation and downregulating pro-inflammatory cytokines [32,33]. Given the deleterious effects of secondary bile acids, particularly DCA, in driving intestinal permeability defects and immune dysregulation, the ability of *P. eryngii* to regulate tight junction integrity and counteract inflammatory signalling suggests a potential protective role against DCA-induced barrier dysfunction. However, the precise mechanisms through which *P. eryngii* mitigates bile acid-induced gut permeability defects remain poorly characterized.

Thus, the aim of the present study was to investigate the ability of *P. eryngii* mushroom fermentation supernatant (FS) to counteract DCA-induced intestinal hyperpermeability and to explore its immunomodulatory properties and effects on signalling pathways, incorporating both an *in vitro* Caco-2 cell culture model and an *ex vivo* system with human colonic tissues.

## 2. Materials and Methods

### 2.1. Source of P. eryngii Supernatant

The fermentation supernatants of *P. eryngii* were generated through an *in vitro* static batch culture fermentation conducted over a 24 h period, using faecal samples obtained from five healthy elderly donors (≥65 years old), as previously described [28,34]. The fermentation substrate consisted of 2% (*w*/*v*) lyophilized powder, which was derived from entire fruitbodies (mushrooms) of the *P. eryngii* (PEWS) strain LGAM 216, cultivated on a wheat straw substrate [35,36]. Following fermentation, samples underwent centrifugation at 10,000× *g* for 30 min at 4 °C to separate the supernatants, which were subsequently filtered using a 0.22 µm pore size filter (Millex^®^, Merck KGaA, Darmstadt, Germany) and stored at −80 °C. To assess the effect of FSs *ex vivo* via stimulation of colonic biopsies, the FS of a single was chosen based on its microbial community composition, immunological profile, and SCFA production levels [28].

### 2.2. Cell Culture of Caco-2 Cells and Sodium Deoxycholate (SDC)

The Caco-2 human colon adenocarcinoma cell line was obtained from the American Type Culture Collection (ATCC) and cultured in Dulbecco’s Modified Eagle Medium (DMEM), enriched with stable glutamine (Biosera, Nuaille, France) and supplemented with 1% penicillin/streptomycin (10,000 U/mL, Biochrom AG, Berlin, Germany) and 10% (*v*/*v*) fetal bovine serum (Biochrom AG). Cultures were maintained in a humidified incubator at 37 °C with 5% CO_2_, utilizing cells from passages 20 to 30. To induce a disruption in intestinal barrier permeability, DCA in the form of sodium deoxycholate (SDC, Sigma-Aldrich, St. Louis, MO, USA) was applied during experimental procedures.

### 2.3. Cell Experimental SDC Conditions Standardization Assays

The viability of Caco-2 cells exposed to SDC was evaluated using the 3-(4,5-dimethylthiazol-2-yl)-2,5-diphenyl tetrazolium bromide (MTT) assay [37]. Briefly, Caco-2 cells were cultured in DMEM and seeded onto a 96-well plate, allowing for stabilization over a 24 h period. Subsequently, the cells were treated with varying concentrations of SDC (0.2, 1, and 2 mM) for 30, 60, 90, and 120 min, and the MTT viability assay was performed as previously outlined [27]. Absorbance measurements were recorded at 540 nm using a PowerWave™ XS microplate reader (BioTek Instruments Inc., Winooski, VT, USA), with data collection facilitated by Gen5 software version 2.0 (BioTek Instruments Inc., Winooski, VT, USA). Cell viability was calculated as a percentage relative to untreated controls. All experiments were conducted in triplicates for each condition and repeated at least twice.

Based on the results obtained from the viability assays, we further assessed the effect of SDC on selected TJ (*zonula occludens-1* (*ZO-1*), *occludin*, and *claudin-1* (Table 1) gene expression for concluding the final stimulation conditions. Caco-2 cells were initially seeded in 6-well plates at a density of 300,000 cells per well and in DMEM complete medium overnight. Subsequently, the cells were exposed to SDC (1 mM) for 30, 60, 90, and 120 min at 37 °C in a 5% CO_2_ environment. Total RNA was isolated, employing the NucleoZOL reagent (MACHEREY-NAGEL GmbH & Co. KG, Dueren, Germany), in accordance with the manufacturer’s protocol. Subsequently, quantitative real-time PCR (RT-qPCR) was carried out using the StepOne PCR System (Applied Biosystems, Waltham, MA, USA) and the KAPA SYBR^®^ FAST qPCR Kit (Kapa Biosystems, Wilmington, MA, USA). The thermal cycling conditions consisted of an initial denaturation, an initial phase at 95 °C for 3 min, followed by 40 cycles of 95 °C for 15 s and 60 °C for 1 min. To quantify transcript levels comparatively, the fold-change analysis was conducted applying the 2^−ΔΔCt^ formula for relative expression measurements.

### 2.4. Treatment of Caco-2 Cells with FSs and SDC

The Caco-2 cells were seeded at a density of 135,000 per well in 6-well plates and incubated overnight in a fully enriched DMEM growth medium at 37 °C in a humidified environment with 5% CO_2_. The upcoming day, the cells were incubated with FS-PEWS of each donor for a period of 48 h at a final concentration of 2% *v*/*v*, as priorly determined [27]. This was followed by an exposure to 1 mM SDC for 90 min under the same controlled conditions. Additionally, a parallel set of cells treated exclusively with SDC (cells + SDC) were employed as an internal control to evaluate the direct impact of SDC on intestinal barrier integrity. At the end of the incubation period, cells’ supernatants were collected, centrifuged at 10,000× *g* for 5 min at 4 °C, and stored in −80 °C for further analysis.

### 2.5. Preparation of Specimens for TJ Gene Expression Analysis

Following treatment from each donor, cells were collected for RNA isolation, which were then processed for RT-PCR to quantify the expression of specific TJ genes, including *ZO-1*, *occludin*, and *claudin-1*, using the same methods as outlined earlier in Section 2.3. The housekeeping gene *β*-actin was used as an internal control, while untreated cells served as the baseline reference. Relative quantification of gene expression was determined using the RQ = 2^−ΔΔCt^ method.

### 2.6. Ussing Chamber Experiment

#### 2.6.1. Study Participants and Ethics

Healthy adult subjects with a median age of 29.7 years (range: 21–62) were recruited through advertisements at Örebro University, Sweden. Participants were excluded if they met any of the following criteria: (a) prior abdominal surgery, (b) hypertension requiring medical management, (c) lactose intolerance, (d) a diagnozed psychiatric disorder, (e) use of prescribed medications or oral contraceptives within 14 days before the study, (f) a history of premenstrual syndrome, and (g) pregnancy or breastfeeding during the study period.

All procedures involving human participants were reviewed and approved by the Regional Ethical Review Board in Uppsala, Sweden (Dnr. 2019–01768), and the study was registered at www.clinicaltrial.gov (ID: NCT05446610). The research was conducted in full compliance with the Helsinki Declaration, and informed written consent was obtained from all participants prior to enrolment.

#### 2.6.2. Collection of Colonic Biopsies

Study participants (*n* = 10) underwent a distal colonoscopy at University Hospital Örebro in the morning after overnight fasting without a bowel cleansing procedure in order to avoid altering the mucosal epithelium. Colonic biopsies were obtained, from the sigmoid colon at the level of the crossing with the common ileac artery, using non-spiked Captura biopsy forceps (Cook Medical, Bloomington, IN, USA) [38], and were immediately transferred to 4 °C oxygenated modified Krebs–Ringer bicarbonate buffer (KRB; aqueous solution with 115 mM NaCl, 1.25 mM CaCl_2_, 1.2 mM MgCl_2_, 2 mM KH_2_PO_4_, and 25 mM NaHCO_3_, set to a pH of 7.2 with 1 M HCl solution and then oxygenated with gas containing 95% O_2_ and 5% CO_2_) and were transported in KRB buffer to the laboratory within 10 min, as previously described [39].

#### 2.6.3. Experimental Procedure

Prior *in vitro* investigations utilizing the Caco-2 cell line (ATCC HTB-37) identified a 2% *v*/*v* concentration of the FS as the most efficacious level for subsequent experimental applications [27]. As part of a pilot assessment, this concentration underwent further evaluation within the *ex vivo* system, where FS-PEWS was assessed at gradient concentrations of 1%, 2%, and 5% *v*/*v* in the KRB buffered medium. Of these, the 2% *v*/*v* concentration exhibited the most significant bioactive effects, prompting its adoption as the standard for subsequent experimental methodologies. For the following experimental setup, two unstimulated biopsies were used as controls, while two biopsies were stimulated only with the SDC stressor, with a final concentration of 1 mM. Two biopsies were stimulated with FS-PEWS (2% *v*/*v*) and two with SDC + FS-PEWS (1 mM + 2% *v*/*v*). Each biopsy was carefully mounted in a 1.5 mL Ussing chamber (Harvard Apparatus Inc., Holliston, MA, USA) between polyester films exposing a 1.77 mm^2^; biopsy area. Biopsies were first equilibrated in ice-cold, oxygenated buffers containing glucose (0.01 M) on the serosal side and mannitol (0.01 M) on the mucosal side. Following a 25 min equilibration, chambers were replenished with 37 °C buffers, and FS-PEWS was added to the mucosal side, followed 20 min later by SDC and markers FITC–dextran 3000-5000 (FD4, Sigma-Aldrich; 2.5 nM) and 45 kDa protein horseradish peroxidase (HRP; Type IV, Sigma-Aldrich; 5.38 μM). As a result, paracellular and transcellular permeability were measured at baseline (T0), and at 60 min (T_60_) and 90 min (T_90_) after the markers were added. All chambers were maintained at 37 °C with constant 95% O_2_ and 5% CO_2_ oxygenation, and the biopsy viability was evaluated via transepithelial electrical resistance (TER), potential difference (PD), and short-circuit current (Isc) readings every 30 s.

Baseline median transepithelial resistance (MTER) values were measured 20 min prior to any treatment to establish a reference point for each donor and to ensure homogeneity between biopsies. Biopsies with uncertain viability were excluded from subsequent analyses based on deviations in TER and PD measurements. Specifically, biopsies were assessed relative to the TER and PD values of other biopsies from the same donor at T_0_. Any biopsy displaying noticeably lower TER values alongside unstable PD measurements, inconsistent with the general range of the donor’s other samples, was considered non-viable and excluded. This donor-specific approach was implemented to account for inter-individual variability and to maintain the reliability of the analyses. After 90 min, biopsies were quickly removed from the chambers and stored in RNALater (Thermo Fisher Scientific, Waltham, MA, USA) at 4 °C overnight and further kept at −80 °C for further RNA analyses.

#### 2.6.4. Measurement of FITC–Dextran and HRP

The FITC–dextran permeability assay was conducted in duplicates, using a standard curve prepared by diluting a FITC solution in glucose KRB, within a 96-well plate format. Fluorescence was quantified at excitation and emission wavelengths of 485 nm and 530 nm, respectively, on an EnSpire Multimode Plate Reader (PerkinElmer, Waltham, MA, USA). For HRP translocation, quantification was performed via an ELISA protocol using the QuantaBlu Fluorogenic Peroxidase Substrate Kit (Thermo Fisher Scientific), following the methodology outlined previously [40]. Briefly, a 96-well plate was coated overnight at 4 °C in the dark with carbonate buffer (pH 9.6; Na_2_CO_3_, Sigma; NaHCO_3_, Merck, Germany) and 5 mg/mL anti-HRP monoclonal mouse IgG antibody (GenWay, San Diego, CA, USA). The next day, the plate was rinsed with PBS-Tween and incubated with 5% BSA blocking buffer (100 mg/mL BSA, PBS, H_2_O) under agitation (500 rpm) in the dark for 1 h. After additional washing, a standard curve and samples prepared in duplicate (100 mg/mL BSA in glucose KRB) were loaded onto the plate and incubated under dark conditions with shaking (300 rpm) for 1 h. Following further washes with PBS-Tween, glucose KRB with 0.2 mg/mL BSA and the substrate solution (H_2_O_2_) were added to each well. After 30 min of dark incubation under shaking (300 rpm), a stop solution was introduced, and after a final 10 min incubation (300 rpm), fluorescence was measured at excitation and emission wavelengths of 315 nm and 470 nm using the EnSpire^®^ Multimode Plate Reader (PerkinElmer, MA, USA). FITC–dextran and HRP passages are expressed as ΔT_90_-T_0_.

### 2.7. Samples Processing for Cytokines and Receptors Gene Expression Analysis

Immune- and signalling- related analysis focused only on high SDC responders, which were biopsies that exhibited at least a 50% increase in both paracellular and transcellular permeability levels after they were treated with SDC and compared with control biopsies (*n* = 5/*n* = 10). RNA extraction from each colonic biopsy was completed using the AllPrep DNA/RNA Micro Kit (Qiagen, Hilden, Germany) according to the manufacturer’s instructions, with quality and concentration assessed as outlined in Section 2.3. A total of 1 ug of RNA was reverse transcribed into cDNA with the High-Capacity cDNA Reverse Transcription Kit (Applied Biosystems, MA, USA). Quantitative real-time PCR (qRT-PCR) was then conducted using the SensiMix™ SYBR^®^ Low-ROX Kit (Nordic Biosite AB, Täby, Sweden) on 96-Well PCR Plates (Sarstedt, Inc., Hildesheim, Germany). The thermal protocol included an initial 10 min denaturation at 95 °C, followed by 40 cycles of 15 s at 95 °C and 15 s at 60 °C. *β*-actin served as the housekeeping gene and untreated biopsies were used as the reference control. Each reaction, conducted in duplicate with 100 ng/uL cDNA and 250 nM primers, targeted cytokine genes (*IL-1β*, *TNF-α*, *IFN-γ*, *IL-6*, *IL-8*, *IL-10*, *IL-13*, *IL-17*) and receptor genes (*dectin-1*, *TLR-2*, *TLR-4*, *NF-kB*, *complement receptor-3* (*CR3*), *mTOR*), as listed in Table 1. The RT-PCR assays were run on a BioRad CFX96 Real-Time System C1600 Touch Thermal Cycler, with data visualized using Bio-Rad CFX Manager version 3.1 (BioRad Laboratories, Hercules, CA, USA). Primer specificity was confirmed via melting curve analysis. Relative transcript quantification was calculated using the 2^−ΔΔCt^ method.

**Table 1:** Sequences of primers used for quantitative real-time PCR.

Gene	Primer Sequences (5′-3′)	Reference
Human *β-actin* F	GCGCGGCTACAGCTTCA	[41]
Human *β-actin* R	CTTAATGTCACGCACGATTTCC	[41]
Human *ZO-1* F	TTCACGCAGTTACGAGCAAG	[42]
Human *ZO-1* R	TTGGTGTTTGAAGGCAGAGC	[42]
Human *Occludin* F	ACAAGCGGTTTTATCCAGAGTC	[42]
Human *Occludin* R	GTCATCCACAGGCGAAGTTAAT	[42]
Human *Claudin-1* F	TGGTCAGGCTCTCTTCACTG	[42]
Human *Claudin-1* R	TTGGATAGGGCCTTGGTGTT	[42]
Human *IL-1β* F	ACAGATGAAGTGCTCCTTCCA	[43]
Human *IL-1β* R	GTCGGAGATTCGTAGCTGGAT	[43]
Human *TNF-α* F	TCTCGAACCCCGAGTGACAA	[44]
Human *TNF-α* R	TATCTCTCAGCTCCACGCCA	[44]
Human *IFN-γ* F	ATCCAGTTACTGCCGGTTTG	[45]
Human *IFN-γ* R	GAAGCACCAGGCATGAAATC	[45]
Human *IL-6* F	AGACAGCCACTCACCTCTTCAG	NM_000600
Human *IL-6* R	TTCTGCCAGTGCCTCTTTGCTG	NM_000600
Human *IL-8* F	GAGAGTGATTGAGAGTGGACCAC	NM_000584
Human *IL-8* R	CACAACCCTCTGCACCCAGTTT	NM_000584
Human *IL-10* F	GGAGAACCTGAAGACCCTCA	[46]
Human *IL-10* R	GATGTCAAACTCACTCATGGC	[46]
Human *IL-13* F	ACGGTCATTGCTCTCACTTGCC	NM_002188
Human *IL-13* R	CTGTCAGGTTGATGCTCCATACC	NM_002188
Human *IL-17* F	CGGACTGTGATGGTCAACCTGA	NM_002190
Human *IL-17* R	GCACTTTGCCTCCCAGATCACA	NM_002190
Human *Dectin-1* F	AACCACAGCTACCCAAGAAAAC	[47]
Human *Dectin-1* R	GGGCACACTACACAGTTGGTC	[47]
Human *TLR-2* F	CTTCACTCAGGAGCAGCAAGCA	NM_003264
Human *TLR-2* R	ACACCAGTGCTGTCCTGTGACA	NM_003264
Human *TLR-4* F	CCCTGAGGCATTTAGGCAGCTA	NM_138554
Human *TLR-4* R	AGGTAGAGAGGTGGCTTAGGCT	NM_138554
Human *NF-kB* F	GGGGATGGTGAGAAGGTTGG	NM_001319226.2
Human *NF-kB* R	GCAGTGCCATCTGTGGTTGA	NM_001319226.2
Human *CR3* F	AAGTCCTCGTTGTCCGTTCC	MW027613.1
Human *CR3* R	CTGCAGCCATTTAACAGCCC	MW027613.1
Human *mTOR* F	GCCGCGCGAATATTAAAGGA	NM_001386500.1
Human *mTOR* R	CTGGTTTCCTCATTCCGGCT	NM_001386500.1

### 2.8. Quantitative Analysis of Cytokine Secretion

The release of cytokines, including IFN-γ, IL-1β, IL-6, IL-10, and TNF-α, was quantified in the supernatants of Caco-2 cells after centrifugation at 10,000× *g* for 5 min at 4 °C. Analyses were performed using the MSD V-Plex Panel (Meso Scale Discovery, Rockville, MD, USA) following the manufacturer’s guidelines. Correspondingly, cytokine levels were measured in serosal fluids derived from colonic biopsies. Samples were diluted at ratios ranging from 1:1 to 1:10, with each sample analyzed in duplicate to enhance reliability. The detection range and sensitivity parameters of the assay are presented in Table S1.

### 2.9. Statistical Analysis

Since no prior data are available on the primary endpoint of this study, the impact of dietary fibre fractions on human intestinal permeability, assessed via FITC-HRP passages, we defined a meaningful effect size as a difference at least equivalent to the standard deviation of the outcome. Furthermore, the anticipated physiological response to the stressor in a healthy population (~60%) was considered in the sample size estimation. Based on a one-way ANOVA with a significance threshold of 5% and a statistical power of 80%, we determined that a minimum of 10 stressor-responsive individuals would be required to detect such a difference with sufficient sensitivity.

The viability data for SDC were determined by calculating the mean of all technical replicates corresponding to each biological replicate. Gene expression levels, including those of TJ, cytokines, and receptors, were quantified by averaging technical replicates across all biological samples for each target gene and normalizing against the corresponding reference gene within each technical run. Permeability assessments, specifically FITC–dextran and HRP passages, were computed by averaging across biological replicates for each treatment condition. The distribution normality of continuous variables was assessed using the Shapiro–Wilk test, complemented by histogram visualizations. Statistical comparisons between the controls and the different treatment groups for each variable were performed, utilizing non-parametric analysis applying the Wilcoxon signed-rank test. All analyses were performed in IBM^®^ SPSS^®^ Statistics, version 28, with significance defined at a 5% threshold (*p* < 0.05).

## 3. Results

### 3.1. Effect of SDC on Cell Viability and Tight Junctions in Caco-2 Cells

**Figure 1:**
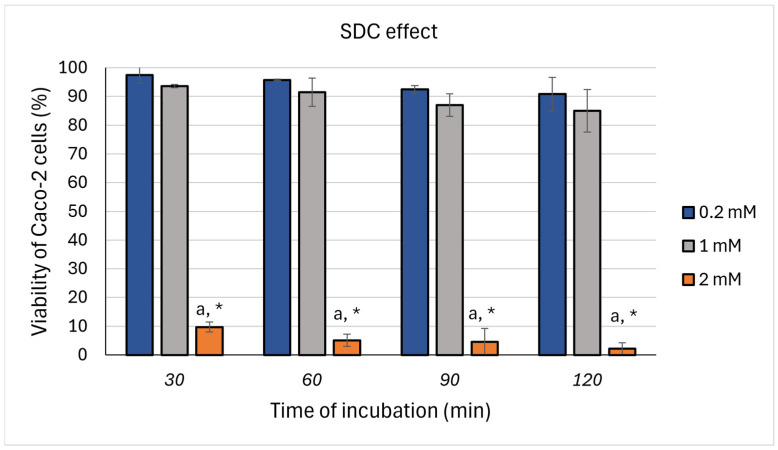
Effects of sodium deoxycholate (SDC) on Caco-2 cell viability (MTT assay). Caco-2 cells were seeded in a 6-well plate and treated with SDC at concentrations of 0.2 mM, 1 mM, and 2 mM for 30, 60, 90, and 120 min. Cell viability is expressed as a percentage relative to the untreated control group, which was set at 100%. The results are presented as mean ± standard deviation of three independent experiments. ^a^ statistically significant compared to 0.2 mM; * statistically significant compared to 1 mM; *p* < 0.05 (Wilcoxon signed-rank test).

The MTT assay was conducted to determine the optimal concentration and incubation duration of SDC in order to challenge the Caco-2 cells since conditions for FS- PEWS treatments have been priorly set [27]. Figure 1 delineates the effect of SDC on Caco-2 cell viability, quantified via the MTT assay across varying concentrations (0.2 mM, 1 mM, and 2 mM) and incubation periods (30, 60, 90, and 120 min). The influence of SDC on cell viability exhibited discernible trends contingent upon concentration and incubation time, revealing time- and dose-dependent responses. At the 0.2 mM concentration, cell viability remained above 90% at all assessed time points (T_30_: 97.47 ± 2.79% to T_120_: 90.81 ± 5.85%), indicating negligible cytotoxicity. Conversely, the 1 mM concentration induced a moderate decline in viability, with values ranging from approximately 93% at T_30_ to 85% at T_120_, yet maintaining over 90% viability after 60 min of exposure (91.52 ± 4.89%). At the highest concentration of 2 mM, a substantial reduction in cell viability was noted, plummeting below 10% after 30 min (9.72 ± 1.78%) and approaching 0% by 120 min (2.17 ± 2.12%). Statistical analyses revealed that the viability at the 2 mM concentration was significantly lower than that at both the 0.2 mM and 1 mM concentrations across all time points, while no significant differences were observed between the 0.2 mM and 1 mM concentrations at any incubation time.

**Figure 2:**
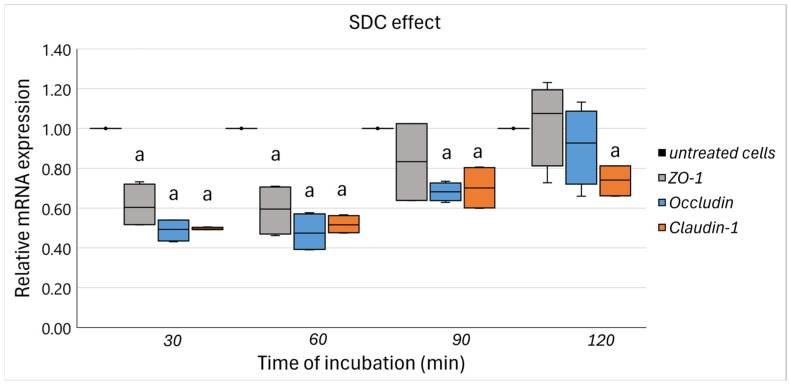
The relative expression of TJ genes in response to SDC-stimulated (1 mM) Caco-2 cells for 30, 60, 90, and 120 min. Data are expressed as mRNA expression (normalized to *β*-actin) relative to untreated cells as mean ± SD of two independent experiments. ^a^ statistically significant compared to untreated cells; *p* < 0.05 (Wilcoxon signed-rank test).

In this context, the relatively mild effect of the 1 mM concentration was identified as the most suitable for subsequent experiments, prompting an additional validation analysis focused on the expression of TJ genes. As illustrated in Figure 2, a U-shaped response was noted over the incubation period for all TJ (*ZO-1*, *occludin*, and *claudin-1*) following treatment with 1 mM SDC. Specifically, the lowest expression levels for all TJ compared to untreated controls were observed after 30 min of incubation (*ZO-1*: 0.61 ± 0.40 (*p* = 0.006), *occludin*: 0.49 ± 0.06 (*p* < 0.001), *claudin-1*: 0.50 ± 0.01 (*p* < 0.001)) and remained diminished even after 60 min. A gradual alteration in this trend was evident after 90 min of incubation, with *ZO-1* expression levels resembling those of untreated cells (0.83 ± 0.22, *p* = 0.228), while *occludin* (0.68 ± 0.05, *p* < 0.001) and *claudin-1* (0.70 ± 0.11, *p* = 0.014) remained significantly reduced, albeit at higher levels than observed at earlier time points. After 120 min of incubation, TJ gene expression appeared to be largely restored and nearly comparable to untreated cells, except for *claudin-1*, which remained significantly decreased (0.74 ± 0.09, *p* = 0.009).

Thus, an incubation period of 90 min at a concentration of 1 mM was identified as the optimal condition for challenging Caco-2 cells with SDC. This parameter was selected to effectively impair intestinal barrier integrity while reducing the risk of severe cytotoxic effects.

### 3.2. FS-PEWS Enhances Tight Junction Genes to Sustain Barrier Integrity Under SDC-Induced Stress in Caco-2 Cells

**Figure 3:**
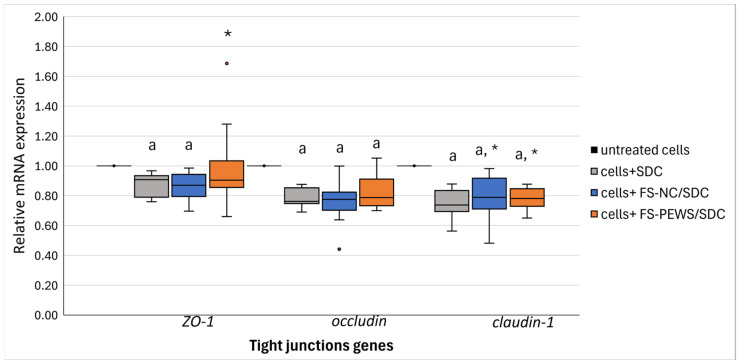
The relative expression of TJ (*ZO-1*, *occludin*, *claudin-1*) genes in response to incubation (2% *v*/*v* for 48 h) with FS-NC and FS-PEWS from a total of five faecal donors in the SDC-stimulated (1 mM for 90 min) Caco-2 cells. Data are expressed as mRNA expression (normalized to *β*-actin) relative to untreated cells as mean ± SD of two independent experiments. Untreated cells: culture cells without any effect, Cells + SDC: culture cells stimulated only with SDC, FS-NC/SDC: culture cells incubated with FSs of the negative control (basal medium with no carbohydrate source) and then stimulated with SDC; FS-PEWS/SDC: culture cells incubated with FSs of *P. eryngii* mushroom and then stimulated with SDC; ^a^ statistically significant compared to untreated cells; * statistically significant compared to SDC; *p* < 0.05 (Wilcoxon signed-rank test).

The efficacy of FS-PEWS in preserving intestinal barrier integrity was evaluated by analyzing the expression levels of TJ genes in Caco-2 cells subjected to SDC stress (Figure 3). Exposure to SDC alone induced a marked downregulation in TJ gene expression compared to untreated cells (*p* < 0.001). On the contrary, treatment with FS-NC yielded a significant increase in *claudin-1* expression [0.76 (0.69, 0.87)] versus levels in cells treated solely with SDC [0.73 (0.59, 0.81)] (*p* = 0.001). However, FS-NC had a less pronounced effect on *ZO-1* [0.88 (0.72, 1.00)] and *occludin* [0.78 (0.71, 0.83)] expression, both of which remained significantly lower than levels in untreated cells (*p_zo-1_* < 0.001, *p_occludin_* < 0.001, *p_claudin-1_* < 0.001).

In contrast, FS-PEWS treatment effectively countered the SDC-induced decline in TJ gene expression. *ZO-1* levels rose significantly in FS-PEWS treated cells [0.89 (0.80, 1.06)], closely approximating those observed in untreated controls (*p* = 0.026). *Claudin-1* expression also recovered substantially [0.78 (0.70, 0.87)], demonstrating a marked increase compared to SDC stressed cells (*p* < 0.001), although it remained below the untreated cells (*p* < 0.001). *Occludin* expression, however, showed limited response to FS-PEWS, displaying levels akin to those of SDC-exposed cells [0.79 (0.71, 0.84), *p* = 0.145] and significantly lower than in untreated controls (*p* < 0.001).

### 3.3. Fermented P. eryngii Mushrooms Preserve Viability of Colonic Specimens

Electrophysiological parameters, specifically TER, PD, and Isc, were methodically assessed over time during the Ussing chamber assays to ensure tissue viability and experimental reliability. As depicted in Figure S1, it was confirmed that biopsies incorporated into the final analysis across different treatment groups demonstrated comparable initial viability characteristics.

For further standardization, TER values were normalized to the initial measurement at T_0_, which was set at 100%, with subsequent readings at T_60_ and T_90_ adjusted accordingly. Analysis of these values indicated that untreated biopsies (median TER: 80.05%, *p*_T90_ = 0.005) and those exposed solely to FS-PEWS (median TER: 81.27%, *p*_T90_ = 0.011) demonstrated notably higher TER maintenance. In contrast, biopsies subjected to the SDC stressor exhibited a significantly lower median TER of 65.29% over the course of the 90 min Ussing chamber experiment (Table S2). Meanwhile, biopsies exposed to SDC following FS-PEWS treatment (median TER: 64.64%) showed comparable viability to those treated with SDC alone but significantly lower TER values relative to untreated or biopsies only treated with FS-PEWS (*p* = 0.007 and *p* = 0.028, respectively) after the 90 min period.

### 3.4. Fermentation Supernatant of P. eryngii Mushrooms Preserve and Attenuate SDC-Induced Increased Paracellular and Transcellular Permeability, Respectively

**Figure 4:**
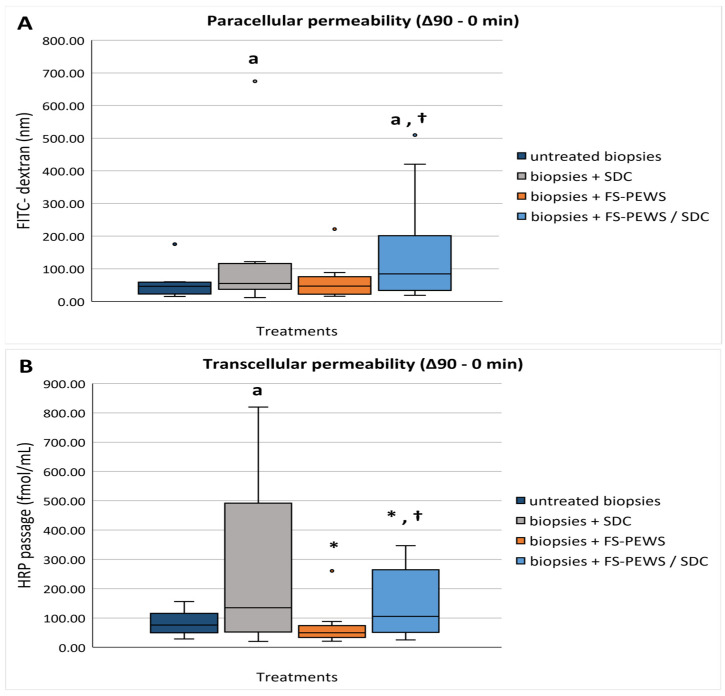
(**A**,**B**) Effects of FS-PEWS, SDC and their co-stimulation on colonic. (**A**) paracellular permeability; (**B**) transcellular permeability in biopsies mounted in Ussing chambers. Data (Δ90-0 min) are presented as a line intersecting the median. Dots represent the outlier values. Untreated biopsies: biopsies without any treatment (*n* = 10); biopsies + SDC: biopsies stimulated only with SDC (*n* = 10); biopsies + FS-PEWS: biopsies incubated only with FS of *P. eryngii* untreated mushroom from one universal faecaldonor (*n* = 10); biopsies + FS-PEWS/SDC: biopsies incubated with FS of *P. eryngii* untreated mushroom from one universal faecal donor and then stimulated with SDC (*n* = 9); ^a^ *p* < 0.05 statistically significant compared to untreated biopsies; * *p* < 0.05 statistically significant compared to SDC; † *p* < 0.05 statistically significant compared to FS-PEWS; Wilcoxon matched-pairs signed-rank test.

To evaluate permeability dynamics, FITC and HRP passages were measured as indicators of paracellular and transcellular permeability, respectively. Exposure to the SDC stressor for 90 min significantly increased both FITC [55.31 nm (40.84, 114.38)] and HRP [175.62 fmol/mL (101.30, 474.25)] passages compared to unstressed control biopsies [FITC: 46.37 nm (24.48, 58.29); HRP: 93.86 fmol/mL (50.79, 129.63)] (*p*_FITC_ = 0.022, *p*_HRP_ = 0.037) (Figure 4A,B). In contrast, pre-stimulation with FS-PEWS for 20 min did not alter permeability, maintaining both FITC [47.01 nm (22.13, 71.31)] and HRP [55.28 fmol/mL (44.85, 69.09)] at similar levels to the unstimulated control biopsies (*p*_FITC_ = 0.575; *p*_HRP_ = 0.139).

Furthermore, FS-PEWS pre-treatment with concurrent SDC exposure resulted in a heightened FITC passage [84.33 nm (34.94, 128.48)], mirroring the SDC-induced increase in paracellular permeability observed in the stressor alone condition, with values significantly elevated relative to both untreated biopsies (*p* = 0.022) and FS-PEWS pre-treatment (*p* = 0.013) (Figure 4A). Conversely, FS-PEWS showed regulatory effects on transcellular permeability, where the HRP passage [106.07 fmol/mL (67.51, 252.25)] remained close to control biopsies levels, with significantly lower levels than in the SDC-only condition (*p* = 0.022), though elevated compared to FS-PEWS alone (*p* = 0.005) (Figure 4B).

### 3.5. FS-PEWS Modulates Epithelial Barrier Integrity and Immune Response Under Stress in Colonic Specimens

**Figure 5:**
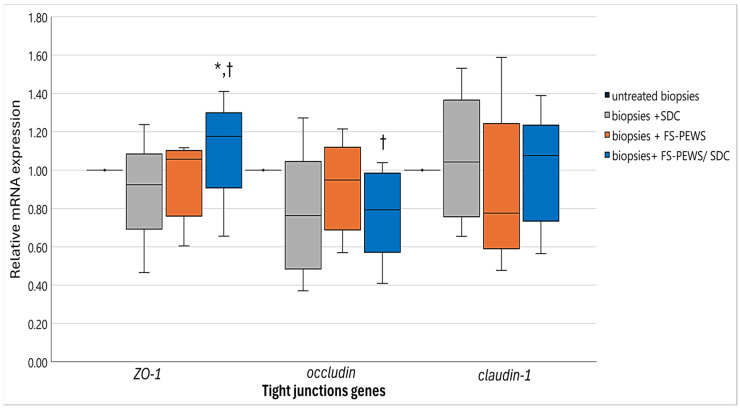
The relative expression of TJ (*ZO-1*, *occludin*, *claudin-1*) genes in response to incubation (2% *v*/*v*) with FS-PEWS with colonic biopsies obtained from a total of five high SDC responders mounted in the Ussing chamber *ex vivo* system. Data are expressed as mRNA expression (normalized to *β*-actin) relative to untreated biopsies as the mean ± SD of two independent experiments. Untreated biopsies: biopsies without any treatment; biopsies + SDC: biopsies stimulated only with SDC; biopsies + FS-PEWS: biopsies incubated only with fermented *P. eryngii* untreated mushroom (2% *v*/*v*) from one universal faecal donor; biopsies + FS-PEWS +SDC: biopsies incubated with fermented *P. eryngii* untreated mushroom (2% *v*/*v*) from one universal faecal donor and then stimulated with SDC; * statistically significant compared to SDC; † statistically significant compared to FS-PEWS; *p* < 0.05 (Wilcoxon signed-rank test).

A subsequent targeted analysis on the colonic biopsies focusing on TJ genes, immunomodulation, and receptor mediated signalling pathways was performed only on the high SDC responders (*n* = 5/*n* = 10). As depicted in Figure 5, the comparison between untreated biopsies and those exposed solely to SDC revealed a relatively unaltered expression of *claudin-1* [1.04 (0.76, 1.37)]. Both *ZO-1* [0.92 (0.70, 1.09)] and *occludin* [0.76 (0.49, 1.05)] showed lower expression levels; however, none of the changes reached statistically significant differences (*p* for all > 0.05).

**Figure 6:**
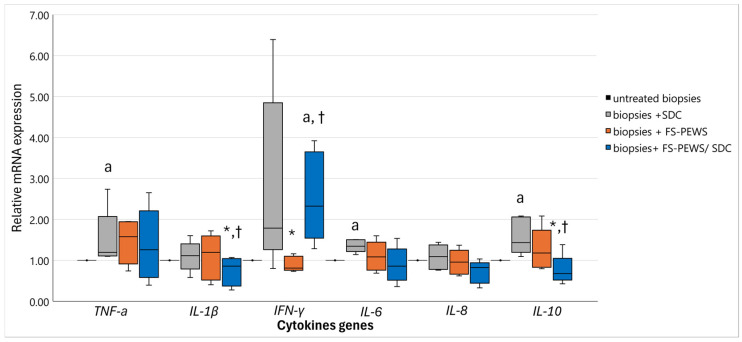
The relative expression of cytokine (*TNF-α*, *IL-1β*, *IFN-γ*, *IL-6*, *IL-8*, *IL-10*) genes in response to incubation (2% *v*/*v*) with FS-PEWS with colonic biopsies obtained from a total of five high SDC responders mounted in the Ussing chamber *ex vivo* system. Data are expressed as mRNA expression (normalized to *β*-actin) relative to untreated biopsies as mean ± SD of two independent experiments. Untreated biopsies: biopsies without any treatment; biopsies + SDC: biopsies stimulated only with SDC; biopsies + FS-PEWS: biopsies incubated only with fermented *P. eryngii* untreated mushroom (2% *v*/*v*) from one universal faecal donor; biopsies + FS-PEWS + SDC: biopsies incubated with fermented *P. eryngii* untreated mushroom (2% *v*/*v*) from one universal faecal donor and then stimulated with SDC; ^a^ statistically significant compared to untreated cells; * statistically significant compared to SDC; † statistically significant compared to FS-PEWS; *p* < 0.05 (Wilcoxon signed-rank test).

Additionally, FS-PEWS treatment alone elicited no noticeable alterations in any TJ gene expression (*ZO-1*: 1.06 (0.76, 1.11), *occludin*: 0.95 (0.69, 1.12), *claudin-1*: 0.78 (0.59, 1.25)) compared to untreated biopsies (*p* for all > 0.05). However, when untreated biopsies were assessed relative to those co-incubated with FS-PEWS and SDC, a nuanced expression pattern was evident. Both *occludin* [0.79 (0.57, 0.99)] and *claudin-1* [1.08 (0.73, 1.24)] remained in line with levels observed in the SDC-only condition (*p* for both > 0.05) but significantly lower when compared to FS-PEWS alone when focusing on *occludin* (*p* = 0.042). On the contrary, *ZO-1* [1.18 (0.91, 1.30)] displayed a substantial expression elevation relative to both SDC (*p* = 0.043) or FS-PEWS (*p* = 0.043) alone. In addition, the cytokine profiles, encompassing *TNF-α*, *IL-1β*, *IFN-γ*, *IL-6*, *IL-8*, and *IL-10*, are depicted in Figure 6. Expression assays for *IL-13* and *IL-17* mRNA were conducted; however, their levels were below the detection threshold and, thus, they were omitted from this report.

Comparatively to untreated ones, biopsies subjected to SDC exposure demonstrated markedly increased expression of *TNF-α* [1.20 (1.11, 2.07), *p* = 0.043], *IL-6* [1.34 (1.22, 1.51), *p* = 0.043], and *IL-10* [1.43 (1.20, 2.06), *p* = 0.043]. An upward trend in *IFN-γ* expression [1.79 (1.26, 4.85)] was also observed, albeit without reaching statistical significance (*p* = 0.080). Notably, SDC exposure did not appear to alter *IL-1β* [1.11 (0.79, 1.40)] or *IL-8* [1.09 (0.78, 1.38)] levels relative to untreated controls (*p* > 0.05). FS-PEWS treatment alone resulted in cytokine expression levels comparable to those in untreated colonic tissues (*p* > 0.05) (Figure 6). When analyzed in relation to the SDC-stimulated biopsies, FS-PEWS treatment induced a significant reduction in *IFN-γ* levels [0.81 (0.76, 1.10), *p* = 0.043], with a similar, though non-significant, downward trend observed for *IL-8* [0.96 (0.66, 1.25), *p* = 0.080]. Upon examining the co-treated samples (FS-PEWS + SDC), FS-PEWS appeared to mitigate the effects of SDC, generally restoring cytokine expression levels to those of untreated tissues, with the exception of *IFN-γ*, which remained elevated [2.32 (1.55, 3.65)] in comparison to both control and FS-PEWS-alone conditions (*p* = 0.043). Moreover, co-treatment resulted in a substantial downregulation of *IL-1β* [0.86 (0.38, 1.05)] and *IL-10* [0.68 (0.52, 1.05)] gene expressions relative to the effects observed with either SDC or FS-PEWS treatment alone (*p* < 0.05). A similar downward trend was noted in *IL-8* levels [0.82 (0.44, 0.94), *p* = 0.080].

**Figure 7:**
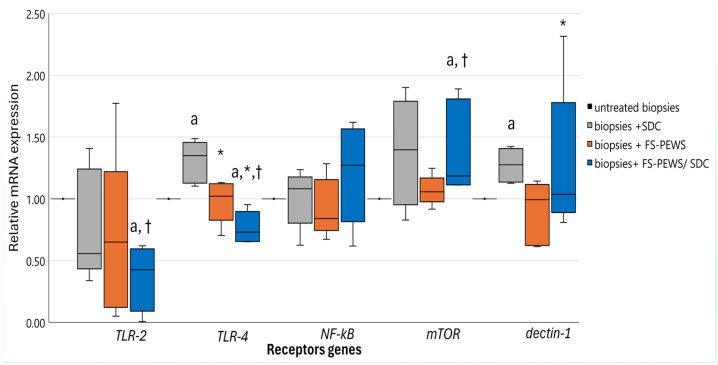
The relative expression of receptors (*TLR-2*, *TLR-4*, *NF-kB*, *mTOR*, *dectin-1*) genes in response to incubation (2% *v*/*v*) with FS-PEWS with colonic biopsies obtained from a total of five high SDC responders mounted in the Ussing chamber *ex vivo* system. Data are expressed as mRNA expression (normalized to *β*-actin) relative to untreated biopsies as mean ± SD of two independent experiments. Untreated biopsies: biopsies without any treatment; biopsies + SDC: biopsies stimulated only with SDC; biopsies + FS-PEWS: biopsies incubated only with fermented *P. eryngii* untreated mushroom (2% *v*/*v*) from one universal faecal donor; biopsies + FS-PEWS + SDC: biopsies incubated with fermented *P. eryngii* untreated mushroom (2% *v*/*v*) from one universal faecal donor and then stimulated with SDC; ^a^ statistically significant compared to untreated cells; * statistically significant compared to SDC; † statistically significant compared to FS-PEWS; *p* < 0.05 (Wilcoxon signed-rank test).

Examination of receptor-mediated signalling pathways revealed distinct activation profiles across treatment conditions, particularly through variations in *TLR-2*, *TLR-4*, *NF-kB*, *mTOR*, and *dectin-1* expression, while *CR3* remained consistently undetectable (Figure 7). In biopsies exposed to SDC, a significant upregulation of *TLR-4* [1.35 (1.13, 1.46), *p* = 0.043] and *dectin-1* [1.28 (1.14, 1.41), *p* = 0.043] was observed in comparison to untreated samples, without further significant modulation in other receptors (*p* > 0.05). In isolation, FS-PEWS treatment had no discernible impact on receptor expression when compared to untreated controls (*p* > 0.05). However, in biopsies preconditioned with SDC, FS-PEWS induced distinct receptor expression changes. Specifically, *TLR-2* [0.43 (0.10, 0.60)] and *TLR-4* [0.73 (0.66, 0.90)] levels were notably reduced relative to both untreated and FS-PEWS-only treated tissues [*TLR-2*: 0.65 (0.12, 1.22); *TLR-4*: 1.02 (0.83, 1.12)], with *TLR-4* significantly downregulated compared to SDC-only conditions (*p* < 0.05). Additionally, *dectin-1* expression was also attenuated [1.04 (0.89, 1.78)] in the SDC + FS-PEWS condition compared to SDC treatment alone (*p* = 0.043). In contrast, FS-PEWS treatment following SDC exposure led to a significant enhancement of *mTOR* expression [1.19 (1.11, 1.81)], exceeding that of both untreated samples (*p* = 0.042) and biopsies only treated with FS-PEWS [1.06 (0.98, 1.17), *p* = 0.043]. Finally, *NF-kB* showed a non-significant trend toward increased expression in co-stimulated tissues [1.27 (0.82, 1.57)] relative to FS-PEWS alone [0.84 (0.75, 1.16), *p* = 0.080].

### 3.6. Absence of Detectable Cytokine Secretion in Both In Vitro and Ex Vivo Systems

An advanced high-sensitivity V-PLEX assay was applied. This analysis targeted the quantification of pro- and anti-inflammatory cytokines, including IFN-γ, IL-1β, IL-6, IL-10, and TNF-α, in both the supernatants derived from Caco-2 cell cultures and the serosal fluids collected from human colonic biopsies. Despite the experimental conditions, neither FS-PEWS treatment nor stimulation with SDC, used as a bile acid stressor, induced detectable cytokine secretion within the assay’s sensitivity thresholds. Furthermore, protein release in the colonic tissue was not further evaluated due to the limitations of the available extraction kits, which were incompatible with simultaneous RNA, DNA, and protein extractions from the same tissue samples.

## 4. Discussion

The GI tract serves as a crucial interface for systemic health, with its selective permeability tightly regulated by epithelial integrity, the resident microbiota, and coordinated immune responses. This intricate system is highly responsive to environmental factors, particularly dietary components, which significantly affect gut health and systemic immunity [48]. Thus, the intestinal barrier is often compromised by endogenous and exogenous agents, notably bile acids. Deoxycholic acid (SDC), a secondary bile acid formed through microbial conversion, is recognized for disrupting epithelial cohesion via cytotoxic and pro-inflammatory effects [13]. Beyond its designation as a bile acid, SDC exerts a diverse array of effects on intestinal epithelial cells, modulating cellular viability, apoptotic processes, the structural fidelity of tight junctions, fluid homeostasis, mucosal secretion, and the induction of pro-inflammatory cytokines [49,50,51]. Moreover, SDC is a potent driver of oxidative stress and genomic instability, pathways intricately linked to oncogenesis. Clinical investigations have consistently reported elevated faecal concentrations of SDC in individuals with ulcerative colitis, dysplastic lesions, and colorectal carcinoma, implicating high SDC levels as a critical risk factor for colorectal cancer development [52]. These insights underscore the value of SDC as a sophisticated experimental model, encapsulating the intricate pathological pathways contributing to epithelial barrier disruption and inflammation on both localized and systemic scales. To the best of our knowledge this is the first study that aimed to investigate the efficacy of *Pleurotus eryngii* mushroom fermentation supernatant (FS-PEWS) in the context of addressing SDC-induced perturbations in intestinal barrier function and immunomodulation, in an *ex vivo* human disrupted colonic tissue model.

To comprehensively elucidate this phenomenon, we utilized a dual methodological framework encompassing both an *in vitro* Caco-2 cell line model and an *ex vivo* Ussing chamber apparatus incorporating human colonic biopsies obtained from healthy adults. This bifocal approach facilitated the evaluation of FSs’ effects under both rigorously controlled and physiologically relevant environments, thereby illuminating both mechanistic pathways and translational implications. Initially, the Caco-2 cell model functioned as a controlled environment to delineate critical experimental parameters, including the concentration of SDC and the duration of incubation. Our findings revealed that the exposure of Caco-2 cells to SDC induced a dose- and time-dependent detriment effect to epithelial viability. SDC is known to induce increased paracellular permeability and instigate cytoskeletal reorganization at the TJ level, further modulating their expression [53,54]. In our study, the application of 1 mM SDC to Caco-2 cells over time elicited a mild U-shaped response in the expression of *ZO-1*, *occludin*, and *claudin-1*. This was marked by an initial decrease in TJ gene expression after 30 min of incubation, followed by a gradual restoration by the 120 min mark, albeit with *claudin-1* expression remaining significantly attenuated in comparison to the control. In related studies, Chen et al. observed a similar U-shaped response in transepithelial electrical resistance (TEER) after 30 min of Caco-2 cells exposure to SDC at concentrations up to 1.6 mM, indicating complex TJ reorganization dynamics [55,56]. Thus, 90 min incubation with 1 mM SDC was confirmed as the optimal sub-lethal exposure condition for subsequent experimental investigations, balancing biological relevance with physiological applicability [6]. While TEER is a widely used functional measure of barrier integrity [57], its omission in our Caco-2 experiments does not compromise the study’s conclusions, as barrier function was directly assessed in the *ex vivo* colonic tissue model.

Dietary composition is pivotal in shaping the gut microbiome and its associated functions, especially in the context of intestinal barrier integrity. A diet abundant in fibre and bioactive compounds, such as those found in mushrooms, offers a strategic approach for promoting gastrointestinal health [58]. *P. eryngii* mushrooms have demonstrated prebiotic potential, fostering a beneficial gut microbiota profile that supports both the integrity of the intestinal barrier and modulates immune functions, as previously substantiated [28,32,59]. The investigation into key TJ gene expression in Caco-2 cells subjected to SDC-induced stress revealed that FS-PEWS is crucial for maintaining intestinal barrier integrity. Exposure to SDC predictably led to a marked reduction in TJ gene expression. Yet, incubation with FS-PEWS effectively countered this decline, restoring *ZO-1* and *claudin-1* expression to levels closely approximating those of untreated cells, although *occludin*’s recovery was comparatively modest. Notably, while the effect of SDC alone was relatively consistent across replicates, greater variability emerged in FS-PEWS and FS-NC conditions, an expected outcome given the use of faecal fermentation supernatants from five distinct donors, each contributing a unique microbial composition and metabolite profile, thereby differentially influencing cellular responses. This affirms *P. eryngii*’s extensive capacity to protect against epithelial barrier disruption under conditions of cellular stress, a capability also increasingly documented in LPS-stimulated *in vitro* models [27,28].

Building upon the promising *in vitro* results, the use of healthy human colonic tissue in the *ex vivo* Ussing chamber setup enabled for comprehensive investigation in a model that maintains the tissue’s native cellular complexity and layered structure. This approach allowed us to assess real-time electrophysiological properties, such as transepithelial electrical resistance (TER) and ion fluxes, providing physiological readouts of tissue viability and barrier functionality [60]. The initial viability of biopsies across various treatment groups was consistent, indicating that any observed electrophysiological alterations could be reliably attributed to the specific interventions, such as a SDC exposure challenge or FS-PEWS treatment. Notably, biopsies treated solely with FS-PEWS exhibited a significant preservation of tissue resistance compared to those subjected to SDC validating further the safety of the selected *P. eryngii* concentration on colonic tissue integrity.

Additionally, the permeability dynamics that we assessed showed that the exposure to SDC markedly increased both FITC and HRP passages compared to control biopsies, reflecting enhanced permeability with the strongest effect being detected on the transcellular passage. This aligns with pre-existing established research that identifies the intracellular space as the main intestinal transportation pathway of SDC due to its size [61]. However, SDC effect on both paracellular and transcellular passages has been described before by Zeng et al. who demonstrated that even a minimal concentration of 0.25 mM SDC induced significant increases in permeability within Caco-2 monolayers, as evidenced through TEER measurements [6]. Meanwhile, FS-PEWS treatment appeared to exhibit a selective modulation, preferentially stabilizing transcellular permeability in the presence of SDC. This stabilization was resembled by a reduced HRP passage, indicative of restored transcellular transport dynamics. Contrastingly, FS-PEWS pre-treatment did not mitigate the SDC-induced increase in paracellular permeability, as reflected by FITC passage measurements, which may suggest a selective functional action of FS-PEWS. This observation could also be attributed to the substantial generation of metabolites, particularly SCFAs, during the 24 h fermentation of *P. eryngii* mushrooms [28]. SCFAs are recognized for their ability to penetrate cells, thereby exerting systemic effects while indirectly modulating paracellular transport pathways [62,63].

Building upon the favourable outcomes of FS-PEWS in preserving intestinal barrier integrity, a targeted analysis of colonic biopsies was undertaken, focusing on TJ gene expression, immunomodulation, and receptor-mediated signalling pathways. We targeted the investigation to individuals classified as high SDC responders to enhance the precision of our findings, despite the small sample size. Their pronounced permeability alterations offer a more robust platform for evaluating FS-PEWS’s efficacy in modulating barrier function and immune responses under stress-amplified conditions. The investigation unveiled discernible regulatory patterns in TJ gene expression, highlighting considerable interindividual variability in gene expression dynamics alongside a subtle impact from both SDC and FS-PEWS treatments. These observations align with the FITC passage analysis, which suggested that the role of the paracellular pathway to barrier dysfunction was not adequately captured within this specific experimental framework.

Specifically, untreated biopsies compared to those exposed solely to SDC showed stability in *claudin-1* mRNA levels, but reductions in *ZO-1* and *occludin*, albeit statistically non-significant, pointed to a potential weakening of epithelial integrity. Similarly, study in murine models administered 250 mg/kg/day SDC via gavage showed negligible impact on TJ gene expression in the proximal colon after 30 min of exposure, with notable effects emerging only after extended treatment (5 days) [55]. Due to rapid tissue viability decline in our *ex vivo* model, extending stimulation beyond 90 min was not feasible. Significantly, FS-PEWS treatment alone did not result in meaningful alterations in TJ gene expression, indicating that there is no disruptive effect on barrier integrity when no external stressors were present. However, a more complex expression pattern emerged under co-incubation with FS-PEWS and SDC. In this dual treatment context, *claudin-1* and *occludin* levels closely mirrored those in samples treated solely with SDC, though *claudin-1* expression was markedly lower than in biopsies treated with FS-PEWS alone. Conversely, *ZO-1* expression was considerably elevated compared to samples treated with either SDC or FS-PEWS alone. These divergent TJ expression responses between the *in vitro* and *ex vivo* models may be attributable to the one-dimension monoculture of Caco-2 cells, which lacks the cellular architecture and intercellular communications present in native tissue, factors that are essential in the intricate regulation of TJ dynamics. This observation underscores the limitations of conventional *in vitro* models, which, while useful as preliminary screening tools, may lack the complexity of more sophisticated multi-layered *in vitro* systems and should ideally be complemented with *ex vivo* systems to enhance result accuracy and reliability.

The assessment of FS-PEWS’s immunomodulatory potential directly in colonic tissue reveals an additional layer of complexity in understanding its impact. Notably, elevated pro-inflammatory cytokines, specifically *TNF-α* and *IL-6*, were observed in response to SDC exposure, consistent with established findings in other models. For instance, murine colonic tissues exposed to 0.2% DCA over 24 weeks led to a substantial increase in *TNF-α*, *IL-6*, and *IL-1β* mRNA levels [64]. Even the upregulation of *IL-10* following SDC exposure likely represents an intrinsic attempt by the colonic tissue to counteract inflammation and restore epithelial integrity [65]. In contrast, the application of FS-PEWS demonstrated an ability to maintain cytokine expression levels akin to untreated controls, suggesting a potential homeostatic role in the absence of inflammatory stimuli. This finding is significant, as it suggests that FS-PEWS may not only preserve intestinal barrier integrity but also modulate immune responses to prevent hyper-inflammatory conditions, a mechanism similarly observed with yeast-derived *β*-glucans, which have been shown to exhibit immunomodulatory properties by regulating inflammatory pathways and enhancing intestinal health [40,66]. Even though there was no no significant reduction in *IFN-γ* levels in SDC-treated biopsies with FS-PEWS, co-treatment suggests a partial re-establishment of immune homeostasis, which is essential for limiting the detrimental consequences of inflammation. Moreover, the co-treatment of FS-PEWS and SDC exhibited a remarkable capacity to mitigate the inflammatory effects induced by SDC, effectively restoring cytokine profiles closer to baseline levels of untreated tissues. In compliance with previous *in vitro* studies this result suggests FS-PEWS as a potential therapeutic agent capable of counteracting the hyper-inflammatory state precipitated by SDC exposure [33]. The downregulation of *IL-1β*, observed in the co-treated biopsies, supports further FS-PEWS’s ability to modulate immune responses toward a more regulated, less inflammatory state, which in turn promotes epithelial integrity and gastrointestinal health [67,68]. The observed cytokine regulation following FS-PEWS exposure, particularly in the presence of SDC, suggests a multifaceted immunomodulatory effect that may involve both direct epithelial signalling and indirect immune interactions [69]. Given that epithelial cells can respond autonomously to environmental stressors [70], FS-PEWS may exert its effects by modulating epithelial receptor activation and downstream transcriptional responses, while also influencing immune-related pathways through its bioactive components. Future studies integrating co-culture models or targeted receptor inhibition assays may help distinguish the primary mechanisms by which FS-PEWS regulates cytokine expression.

Analyses using the high-sensitivity V-PLEX platform demonstrated the absence of detectable protein secretion for cytokines such as IFN-γ, IL-1β, IL-6, IL-10, and TNF-α across all tested experimental systems, despite pronounced changes in their corresponding mRNA expression levels. This discordance between transcriptional and translational outputs likely reflects the intricate temporal kinetics of cytokine biosynthesis, where transcriptional activation occurs earlier than downstream processes of translation and secretion, which may extend beyond the experimental timeframe used in this study [71]. Beyond temporal constraints, this disparity may also stem from post-transcriptional and post-translational regulatory mechanisms that modulate cytokine production at multiple levels [72,73]. mRNA stability, differential splicing, and microRNA-mediated repression are known to affect the efficiency of mRNA translation into protein [74], while post-translational modifications such as glycosylation, phosphorylation, and ubiquitination can regulate protein stability, trafficking, and secretion [75]. Experimental limitations must also be considered. The sensitivity of the V-PLEX assay, while high, has inherent detection limits that may not capture low-abundance cytokines, particularly if secretion is transient or occurs below the detection threshold of the assay. Additionally, the use of a cell culture system lacks the full physiological complexity of the colonic mucosa, which includes immune and stromal interactions that could influence cytokine release dynamics. Future studies employing different incubation times, co-culture models, or proteomic analyses may provide deeper insights into cytokine regulation under these conditions.

This uncoupling of cytokine mRNA expression from protein secretion underscores the complex regulatory mechanisms governing inflammatory responses, many of which are affected by upstream receptor-mediated signalling pathways. These pathways not only influence the regulation of cytokine production but also elucidate key mechanisms of immune modulation. Notably, the upregulation of *TLR-4* and *dectin-1* in biopsies exposed to SDC reinforces the concept of pathogen-associated molecular pattern (PAMP) recognition involvement and subsequent inflammatory activation via Toll-like receptor pathways [76]. The expression of these receptors is intricately linked to the activation of downstream signalling cascades such as NF-kB, which orchestrate the transcriptional activation of pro-inflammatory genes, thus, amplifying the inflammatory response [77]. Evidence has shown that elevated levels of secondary bile acids activate the TLR-4/NF-kB pathway, resulting in cell membrane disruptions and upregulation of cytokines, such as IL-6 and TNF-α [12]. In parallel, FS-PEWS treatment following SDC exposure induced a notable enhancement of *mTOR* expression, surpassing levels observed in both untreated samples and biopsies treated solely with FS-PEWS. This effect was similarly evident in our previous work, where FS-PEWS treatment led to overexpression of *mTOR* in an LPS-stressed Caco-2 model [32]. This finding suggests that mTOR may act as a compensatory mechanism, promoting epithelial repair and cellular homeostasis under conditions of bile acid-induced stress potentially contributing to the attenuation of inflammation observed in the co-treatment condition [78]. Conversely, FS-PEWS treatment, particularly when applied in conjunction with SDC, led to the marked downregulation of *TLR-2* and *TLR-4* in the co-treatment condition suggesting that *P. eryngii* mushrooms may possess the capacity to inhibit receptor-mediated inflammatory signalling, thus, attenuating the excessive immune response elicited by SDC. This is in line with Jiang et al., who demonstrated that polysaccharides from *Grifola frondosa* mushrooms suppressed inflammation in high fat diet-fed mice by modulating the TLR-4/NF-kB pathway, leading to decreased TNF-α, IL-1β, and IL-6 serum levels [79]. While these findings provide initial insights into the modulation of receptor-mediated inflammatory pathways by FS-PEWS, further studies incorporating detailed molecular analyses of downstream signalling events are needed to fully elucidate the mechanistic interactions driving these effects.

In light of the study’s outcomes, it is imperative to highlight the significant inter-individual variability among biopsy donors, a factor of critical importance in the context of “precision nutrition”. This variability underscores the necessity for personalized dietary interventions. While these differences likely reflect intrinsic variability in colonic tissue responses, potential interactions with the microbiota cannot be entirely excluded. However, given that all biopsy samples were exposed to the same FS-PEWS, the observed variability is more likely driven by host-specific factors rather than differences in microbial composition. The findings suggest that incorporating *P. eryngii* mushrooms into dietary practises could serve as a beneficial strategy to support gut health, particularly in individuals predisposed to dysbiosis and inflammation associated with elevated microbial bile acid production. The advantageous effects of FS-PEWS appear to stem, in part, from its unique bioactive compounds, including SCFAs present in the fermented supernatant, which may contribute anti-inflammatory and cytoprotective properties that help alleviate the detrimental effects of bile acid exposure [28]. Future studies integrating individualized microbiota profiling alongside colonic tissue responses may further refine these insights and help disentangle host-driven variability from microbiota-mediated effects.

## 5. Conclusions

In conclusion, the present study unveils groundbreaking insights into the multifaceted potential of the *Pleurotus eryngii* mushroom fermentation supernatant (FS-PEWS) as a promising dietary strategy in mitigating disruptions to intestinal barrier integrity and immune homeostasis. By synergistically assessing both *in vitro* Caco-2 cell models and *ex vivo* human colonic tissue models via the sophisticated Ussing chamber system, we have elucidated a dual protective capacity of FS-PEWS, specifically its remarkable ability to preserve tight junction integrity while concurrently modulating both paracellular and transcellular permeability under the stress of secondary bile acid exposure. Beyond its ability to maintain epithelial barrier structure and function, FS-PEWS has demonstrated a potent anti-inflammatory efficacy during cytokine dysregulation, which was manifested through the downregulation of pivotal pro-inflammatory mediators e.g., *TNF-α,* IL-6, and *IL-1β* and re-establishing immune equilibrium by inhibiting key pattern recognition receptors such as *TLR-4* and *dectin-1*. These findings herald a novel mechanistic paradigm for the therapeutic application of products based on *P. eryngii* mushrooms, positioning them as a highly promising approach for the clinical management of intestinal permeability disorders while also mitigating systemic inflammatory cascades. Thus, this research paves the way for future translational studies, above all *in vivo* models and intervention studies, to further refine our understanding of the specific bioactive constituents within FS-PEWS that underpin these protective effects, opening new avenues for the clinical management of conditions linked to intestinal barrier dysfunction, chronic inflammation, and gut dysbiosis.

## Data Availability

The original contributions presented in this study are included in the article/Supplementary Materials. Further inquiries can be directed to the corresponding author.

## Supplementary Materials

The following supporting information can be downloaded at: https://www.mdpi.com/article/10.3390/nu17040694/s1, Table S1: Concentration detection level of cytokines of V-Plex panel (pg/mL); Figure S1. Median baseline TER (mTER) for the different treatment groups. Boxplots show mTER with the marked median, and whiskers visualize minimum and maximum values.; Table S2. Transepithelial resistance [TER units (%)] values at 60 and 90 min normalized to each participant’s respective 0 min value (*n* = 10).
