# Supplementary material for: Pleurotus eryngii Mushrooms Fermented with Human Fecal Microbiota Protect Intestinal Barrier Integrity: Immune Modulation and Signalling Pathways Counter Deoxycholic Acid-Induced Disruption in Healthy Colonic Tissue
Source: Nutrients. 2025 Feb 14;17(4):694. doi: 10.3390/nu17040694 (PMC11858169; doi:10.3390/nu17040694)
Supplement: Supplementary file 1 [file nutrients-17-00694-s001.zip › nutrients-3454094-supplementary.pdf]

Table S1. Concentration detection level of cytokines of V-Plex panel (pg/mL)

Analyte	Range of measurement (pg/mL)
IFN-g	39.9 – 556
IL-1β	11.2 - 225
IL-6	8.13 – 271
IL-10	3.80 – 134
TNF-α	1.77 – 76.3

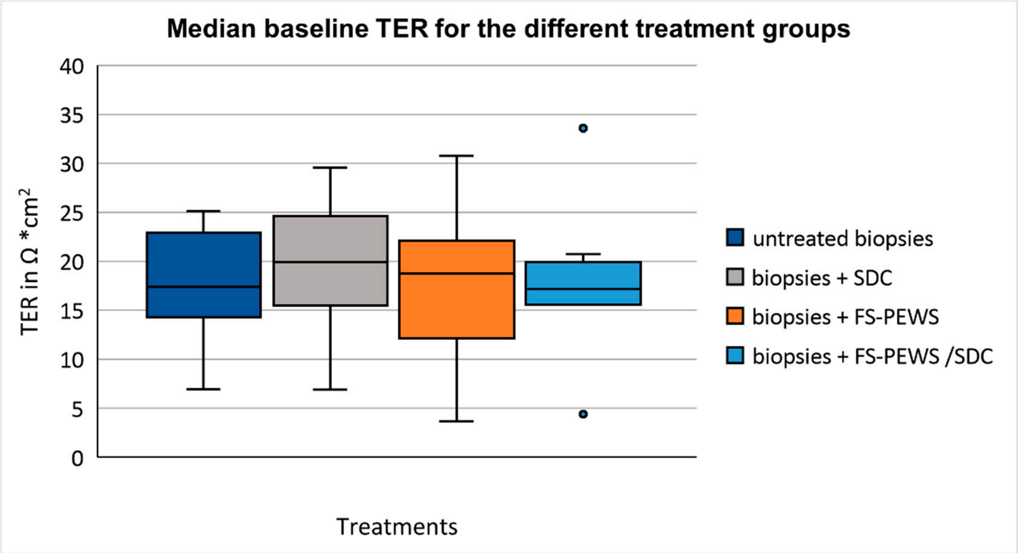

Figure S1. Median baseline TER (mTER) for the different treatment groups. Boxplots show mTER with the marked median, and whiskers visualize minimum and maximum values. Dots represent the outlier values. mTER was determined within a time period of 10 min (T_{-30} to T_{-20}) prior to any treatment or stimulation of colonic biopsies mounted in Ussing chambers (*n*=10). Untreated biopsies: biopsies without any treatment; biopsies + SDC: biopsies stimulated only with SDC; biopsies + FS-PEWS: biopsies incubated only with FS of *P. eryngii* untreated mushroom from one universal fecal donor; biopsies + FS-PEWS/ SDC: biopsies incubated with FS of *P. eryngii* untreated mushroom from one universal fecal donor and then stimulated with SDC; Wilcoxon signed-rank test;

Table S2. Transepithelial resistance [TER units (%)] values at 60 and 90 minutes normalised to each participant's respective 0 min value (*n*=10).

	0 min	60 min	90 min
**Untreated biopsies**	100.0	82.35 (74.76, 88.74)	80.05 (69.20, 87.20)
**Biopsies + SDC (1mM)**	100.0	73.29 (69.09, 76.59) a	65.29 (59.68, 72.92) a
**Biopsies + FS-PEWS (2% v/v)**	100.0	82.54 (77.96, 87.56) *	81.27 (71.75, 86.85) *
**Biopsies + FS-PEWS (2% v/v) / SDC (1mM)**	100.0	74.45 (63.14, 79.75) a, †	64.64 (59.24, 77.41) a, †

Values are expressed as median (interquartile range); TER at the beginning of the experiment (T_0) was set as 100%, and values for T_{60} and T_{90} were normalized to T_0; Untreated biopsies: biopsies without any treatment; biopsies + SDC: biopsies stimulated only with SDC; biopsies + FS-PEWS: biopsies incubated only with FS of *P. eryngii* untreated mushroom from one universal fecal donor; biopsies + FS-PEWS/ SDC: biopsies incubated with FS of *P. eryngii* untreated mushroom from one universal fecal donor and then stimulated with SDC; [a] $p<0.05$ statistically significant compared to untreated biopsies; [*] $p<0.05$ statistically significant compared to SDC; [†] $p< 0.05$ statistically significant compared to FS-PEWS; Wilcoxon matched-pairs signed rank test;
